# Intelligent Wireless Sensor Network Sensor Selection and Clustering for Tracking Unmanned Aerial Vehicles

**DOI:** 10.3390/s25020402

**Published:** 2025-01-11

**Authors:** Edward-Joseph Cefai, Matthew Coombes, Daniel O’Boy

**Affiliations:** Department of Aeronautical and Automotive Engineering, Loughborough University, Loughborough LE11 3TU, UK; d.j.oboy@lboro.ac.uk

**Keywords:** Wireless Sensor Networks, Unmanned Aerial Vehicle, sensor selection, predicted posterior distributions, Extended Kalman Filter

## Abstract

Sensor selection is a vital part of Wireless Sensor Network (WSN) management. This becomes of increased importance when considering the use of low-cost, bearing-only sensor nodes for the tracking of Unmanned Aerial Vehicles (UAVs). However, traditional techniques commonly form excessively large sensor clusters, which result in the collection of redundant information, which can deteriorate performance while also increasing the associated network costs. Therefore, this work combines a predictive posterior distribution methodology with a novel simplified objective function for optimally identifying and forming smaller sensor clusters before activation and measurement collection. The goal of the proposed objective function is to reduce network communication and computation costs while still maintaining the tracking performance of using far more sensors. The developed optimisation algorithm results in reducing the size of selected sensor clusters by an average of 50% while still maintaining the tracking performance of general traditional techniques.

## 1. Introduction

In recent years, Wireless Sensor Networks (WSNs) have been widely adopted in the field of target tracking. Since WSNs are generally made up of low-cost and low-accuracy passive sensors, sensor nodes are commonly clustered to improve the tracking performance of the network. For example, WSNs can be used to estimate the path and future position of Unmanned Aerial Vehicles (UAVs) when ensuring security across a protected area.

In this work, WSN applications for Unmanned Traffic Management (UTM) are considered, where non-battery powered nodes are assumed within a connected WSN. In UTM systems, sensor nodes are used to track and maintain contact with cooperative UAVs, whose locations and trajectories are both known and planned [1]. However, due to the increased usage of civilian UAVs across both urban and rural locations, it is quite easy for non-cooperative UAVs to enter restricted UTM airspace. Thus, it is vital for multiple sensors within UTM systems to be able to detect, track and avoid non-cooperative UAVs in an efficient manner [2].

Traditionally, dynamic techniques cluster nodes by activating all nodes believed to have the estimated position of the target within their ranges [3]. However, the redundant nodes in these strategies may provide unnecessary information and sometimes even degrade the tracking performance. Moreover, these nodes contribute needless communication (comms) and computation costs. Alternatively, techniques such as [4] select nodes closest to the target to increase the probability of collecting a measurement, with the assumption of an accurate target prediction. Furthermore, in some methods, the size of the sensor node selection area increases according to the error of the target estimate [4,5] or the estimated trajectory [6], which can omit potentially useful sensors from the selected sensor node cluster.

It has been identified that for most target-tracking sensor selection solutions, the objective functions developed for guiding the sensor selection processes focus heavily on the constraints and limitations presented by WSNs, such as energy consumption, load balancing, and network lifetime. By focusing on these, the important WSN objective of accurate target tracking is frequently omitted from the sensor selection decision process. The review paper [7] further illustrates the dominance of considering energy usage during the sensor selection process, while the review paper [8] indicates that when tracking quality is considered, energy cost is often ignored.

Therefore, this work proposes a novel target-tracking sensor selection objective function that considers both the expected quality of the sensor information (the sensor utility) as well as the cost of obtaining the information (the sensor cost) for different combinations of the available sensor nodes. The goal of this work is to select the smallest sensor node combination possible while still maintaining tracking performance that comes from using far more nodes.

Moreover, this work has been designed as a generic framework and can be used in conjunction with different sensing models and techniques such as acoustic [9], optical [10,11], radar [12,13], and radio frequency [14] sensors, as well as with two-stage state estimators such as the Extended Kalman Filter (EKF) and the Particle Filter (PF).

To do this, the utility function will measure and compare the uncertainty in predicted posterior distributions for different sensor combinations acquired using predicted measurements for each sensor. Moreover, to establish a combination’s sensing cost, its energy consumption for both communication and computation will be approximated.

Therefore, in summary, the novel contributions of this work include the development of a WSN sensor selection objection function which:

- focuses on the WSN objective of efficient tracking with applications in UTM rather than the WSN constraints;
- considers both the information utility and information cost of different sensor combinations as part of the cluster formation process;
- is designed to be a generic framework and can be applied to numerous types of WSNs, sensor models and two-stage state estimators.

Thus, this paper is structured as follows. Section 1.1 first defines the notations and operators used throughout this work. Section 1.2 first defines the requirements for the objective function, while Section 1.3 and Section 1.4 detail existing techniques for calculating the information utility and cost values, respectively. Section 2.1 provides a detailed methodology for calculating the predicted posterior distribution of a sensor combination using a predicted sensor likelihood model. Section 2.2 and Section 2.3 then detail the formulation for the developed objective function. Lastly, Section 3 discusses the results and performance of the objective function in identifying the sensor combination with the global minimum.

### 1.1. Notations and Operators

The important notations and operators used throughout this work are detailed in Table 1. Please note that boldface script is used to notate vectors and matrices. When first defined in the text, matrices will be notated as $M_{m\times n}$, indicating a matrix with *m* rows and *n* columns.

### 1.2. Objective Function Requirements

The goal of this work is to reduce computation and communication costs across a WSN while still maintaining tracking performance. Therefore, the objective function is required to consider both a sensor combination’s ‘quality of information’, otherwise referred to as its ‘utility’, as well as the combination’s associated costs for obtaining, sharing and processing the sensed information [15].

These individual measures of ‘utility’ and ‘cost’ are common in the field of WSN sensor management, and thus, literature tends to follow a generic equation when forming a sensor selection objective function [16,17,18,19,20]. The objective function, $M\left(p\left(\hat{x}|z_{1},\ldots ,z_{j}\right)\right)$, is commonly formulated as:

(1) $$\begin{array}{c} M\left(p\left(\hat{x}|z_{1},\ldots ,z_{i}\right)\right)=\alpha \cdot \Psi_{utility}\left(x|z_{1},\ldots ,z_{i}\right)-\left(1-\alpha \right)\cdot \Psi_{cost}\left(z_{i}\right) \end{array}$$

where $p\left(\hat{x}|z_{1},\ldots ,z_{i}\right)$ is the probability distribution of the target state estimate ${\hat{x}}_{4\times 1}$ given measurements $z_{1},\ldots ,z_{i}$, $\Psi_{utility}$ is the information utility measure for using measurements $z_{1},\ldots ,z_{i}$, $\Psi_{cost}$ is the cost measure for using sensor *i*, and α is used to assign a relative weighting to the sensor utility and cost measures.

The presence of the weighting value α is necessary as it plays a role in assigning an importance level to the utility and cost measures. Should the utility measure require greater importance than the associated costs measure, an α value in the range of 0.5≤α≤1 should be selected. The higher the α value is, the more importance the information utility measure is given.

### 1.3. Information Utility Measures

Since the optimal sensor selection problem for multiple sensors generates numerous predictions for the target posterior density, methodologies for determining the next optimal sensor set are required.

When predicting the optimal sensor combination for tracking a target at time step *k*, the expected utility of the combination in question must be examined. For this, a sensor combination’s predicted posterior distribution, which illustrates the compactness of its estimate uncertainty as a probability before a measurement has been made, will be the metric available for analysing and comparing each combination’s utility. However, comparison between multiple large distributions can be costly and time-consuming, and therefore, a more condensed utility metric is required for this task. Here, the literature indicates that multiple information-theoretic measures have been applied to determine the utility, or ‘goodness’ of a distribution as a single-value metric that can be easily compared to others.

For example, in [16], Shannon Entropy and Mahalanobis Distance [21] are two different information-theoretic measures used to determine the information utility value of the predicted target posterior distributions for different sensors. Furthermore, in [22], cross-entropy measures the difference between two distributions by assessing the probability of the target estimate distribution against the predicted posterior distribution. Alternatively, in [23], Renyi Information Divergence measures the amount of information a predicted observation can provide to an existing target distribution, whereas in [24], the best sensor set is calculated using a Fisher Information Matrix (FIM) technique in an ‘add one sensor node at a time’ methodology.

By contrast, in [25], an error ellipsoid is extracted from the estimate covariance, where a bearing-only observation is assumed at the distribution’s mean for each sensor. A utility function then calculates each sensor’s information gain based on the distributions’ overlap. A similar methodology is provided in [26], conveying the benefit of not requiring the calculation of multiple posterior distributions. However, using low-quality, high-noise sensors may result in the noise model area being larger than the estimated error, thus making the utility function null.

Therefore, due to its fast and simplistic nature of providing a comparable single-value measure of compactness for a distribution, Shannon Entropy has been selected as the information utility measure.

### 1.4. Sensor Cost Measures

The sensor cost refers to the cost of obtaining sensing information and can be characterised by measures of the node-node communication links and the node energy consumption [16]. These two characteristics are the main aspects of WSN operations, which have the highest energy consumption [27] and are proportional to the number of activated nodes [28]. Furthermore, these two characteristics are in line with the research goals of selecting as few sensors as possible in order to reduce communication and computation costs to allow for the use of low-cost sensor nodes. Thus, it is these two measures that require optimisation during the sensor selection task [29].

As a result of this, WSN sensor selection cost functions generally consider both the number of communications and computations required to perform a sensing task, weighted by application-determined parameters [29]. Equation (2) sets out the generic baseline for a sensor selection cost function,

(2) $$\begin{array}{c} \Psi_{Cost}=N_{Comms}\times C_{Comms}+N_{Comps}\times C_{Comps} \end{array}$$

where $\Psi_{Cost}$ is the sensing cost, $N_{Comms}$ and $N_{Comps}$ are the number of communications and computations, respectively, and $C_{Comms}$ and $C_{Comps}$ are application-determined parameters reflecting the costs of communication and computation for active nodes, respectively.

The following sub-sections each further describe the formulation for the communication and computational cost measures, respectively.

#### 1.4.1. Communication Cost Measures

The communication cost of a sensor refers to quantifying the expense of transmitting and receiving information between two nodes. Since the power to transmit information between two nodes is proportional to the distance between the two nodes, the energy model is frequently used to estimate a rough order of magnitude for the amount of energy consumed to transmit and receive information [20,27,30,31,32,33,34,35,36,37]. Please note that this follows the assumption of direct communication between member nodes and the cluster head. However, Equation (2) allows for other communication topologies, such as mesh networking, to be considered using a different communication cost model.

Using the energy model, the energy cost to transmit ($E_{t}$) and receive ($E_{r}$) *l* bit data to a node at a distance of *d* metres is calculated using Equations (3) and (4), respectively.

(3) $$\begin{array}{c} E_{t}=l\varepsilon_{elec}+l\varepsilon_{amp}d^{4} \end{array}$$

(4) $$\begin{array}{c} E_{r}=l\varepsilon_{elec} \end{array}$$

where $\varepsilon_{elec}$ is the energy per bit to run the electronics, commonly equal to $0.5\times 10^{-7}$ J/bit, $\varepsilon_{amp}$ is the energy per bit to operate the transmission amplifier, commonly equal to $0.13\times 10^{-14}$ J/(bit·m^4^) and *d* is the transmission distance.

In [27], the transmission energy cost for *l* bits of data has been modified to consider and allow the use of varying transmission channel characteristics, where the $d^{4}$ from Equation (3) has been replaced with $d^{\beta }$.

However, as previously stated, since this work considers applications in UTM, thus assuming mains-powered nodes, an energy consumption metric is not required. Instead, a metric that quantifies communication costs as a resource load is more beneficial. Therefore, it is acceptable for the above energy consumption equations to be simplified and considered by just their distance, as this will still be somewhat proportional to the energy consumption costs and still provide a reasonable communication cost with lesser calculation requirements.

For example, in [4,16,17,38], the cost per bit, $C_{ij}$, for direct communication between two nodes *i* and *j*, is calculated as being proportional to the squared Euclidean distance between them, calculated as:

(5) $$\begin{array}{c} C_{ij}\propto \left|\right|y^{i}-y^{j}{\left|\right|}_{2}^{2} \end{array}$$

where $y^{s}$ is the location of the *s*-th sensor. This is also referred to as a simplified model of the energy expense of the radio transmission [17].

Furthermore, in [18], the communication cost model, $C_{i,j}$, is modelled as being proportional to the corresponding distance between the two nodes.

#### 1.4.2. Computation Cost Measures

The computation cost refers to the computational load that is required to calculate the target state estimate. In some work, this has also been calculated as the computational energy consumption.

For example, in [39], the computational energy consumption is calculated as being directly proportional to CPU workload (Equation (7)), defined by the required number of CPU cycles (Equation (6)).

(6) $$\begin{array}{c} W=L\times X \end{array}$$

(7) $$\begin{array}{c} E_{c}=\sum_{w=1}^{W}\epsilon_{c}\left(w\right)=\sum_{w=1}^{W}\kappa f_{w}^{2} \end{array}$$

where *W* is the number of CPU cycles required, *L* is the input data size, *X* is the computation algorithm, $\epsilon_{c}$ is the energy consumption per operation cycles, κ is the effective switched capacity that is determined by the chip architecture, and $f_{w}$ is the scheduled CPU clock frequency for the next CPU cycle given that the number of *w* CPU cycles have been completed [39].

Alternatively, in other work, computational load is defined as the computational complexity of the sensor selection algorithm, which provides a total number of calculation sets completed at each time step. An overview of common algorithm complexities is detailed in [40]. However, the equation most commonly found in the literature refers to the computational complexity of conducting an exhaustive search [20,40], where the number of computations across all possible node combinations, nc, is calculated as:

(8) $$\begin{array}{c} nc=\frac{N_{A}!}{M!\times \left(N_{A}-M\right)!} \end{array}$$

where $N_{A}$ is the number of available nodes, and *M* is the node combination set.

## 2. Formulation and Methodology

In this section, the formulation and methodology for calculating each aspect of the proposed objective function are detailed, including the formulation for the predicted posterior distribution methodology.

### 2.1. Calculating the Predicted Posterior Distribution

This section details the methodology and implementation for predicting sensor measurements, which are used to calculate a predicted posterior distribution for a specific sensor cluster before any measurements have been collected. This will later be used as part of the information utility measure within the objective function to estimate the ‘goodness’ of using specific sensor node combinations to accurately track a target.

This methodology has been implemented in a custom-developed simulation environment and is described using a simple tracking scenario consisting of four bearing-only, omnidirectional, passive sensors and a single UAV target. Each step of the methodology is detailed, using figures, for a single time step. Note that in this example, each sensor is considered a neighbouring node, thus allowing direct communication between nodes. If a sensor node’s range overlaps with the target prior estimate, it can be selected as an active node during that time step.

To calculate a sensor combination’s posterior distribution without the availability of their measurements, this technique first predicts each considered sensor’s measurement likelihood across the current target prior estimate region. Using a modification to Bayes’ rule, the individual sensor likelihoods are then combined and used to predict the posterior distribution for a specific sensor node combination.

The single sensor selection methodology developed in [16] has provided a baseline methodology for this technique. However, this has been modified and is detailed in this section.

#### Mathematical Formulation for Predicting the Posterior Distribution

It is noted that this technique has been developed to use non-parametric beliefs, where target distributions are approximated using discretised grid samples across the region of interest. In line with the aim of this work, this allows for reduced computation over a finite area [41]. However, for ease of understanding and for recognising common equations, some equations have also been defined for continuous data where possible.

*Target Prior Distribution Estimate*

At each time step *k*, the first step is to estimate the target’s prior distribution, $p\left({\hat{x}}_{k}^{-}|z_{k-1}\right)$, using the Chapman-Kolmogorov equation [42,43]:

(9) $$\begin{array}{c} p\left({\hat{x}}_{k}^{-}|z_{k-1}\right)=\int \underset{\mathrm{Posterior}\;\mathrm{from}\;k-1}{\underset{︸}{p\left({\hat{x}}_{k-1}^{+}|z_{k-1}\right)}}\times \underset{\mathrm{Target}\;\mathrm{Dynamics}\;\mathrm{Model}}{\underset{︸}{p\left({\hat{x}}_{k}^{-}|{\hat{x}}_{k-1}^{+}\right)}}d\left({\hat{x}}_{k-1}^{+}\right) \end{array}$$

where ${\hat{x}}_{k_{\;4\times 1}}^{-}$ is the target prior state estimate at time step *k*, $z_{k-1}$ refers to the measurement history until time step k−1 and ${\hat{x}}_{k-1_{\;4\times 1}}^{+}$ is the target posterior state at time step k−1.

However, to allow for faster, lower-cost approximation of the distribution across a finite area, Equation (9) is discretised as:

(10) $$\begin{array}{c} p\left({\hat{x}}_{k}^{-}|z_{k-1}\right)=\sum_{u_{k-1}\epsilon S\left({\hat{x}}_{k-1}^{+}\right)}\left(p\left({\hat{x}}_{k-1}^{+}|z_{k-1}\right){|}_{{\hat{x}}_{k-1}^{+}=u_{k-1}^{a}}\right)\times \left(p\left({\hat{x}}_{k}^{-}|{\hat{x}}_{k-1}^{+}\right){|}_{{\hat{x}}_{k-1}^{+}=u_{k-1}^{a}}\right) \end{array}$$

where $u_{k-1_{\;g_{x}\times g_{y}}}$ is the discretised grid sample space across $p\left({\hat{x}}_{k-1}^{+}|z_{k-1}\right)$ at time step k−1, with sample sizes $g_{x}$ and $g_{y}$ in the x- and y-directions respectively, and $u_{k-1}^{a}$ refers to grid sample *a* in $u_{k-1}$.

The implementation of the prior estimate (Equation (10)) is made using the prediction stage of the Extended Kalman Filter (EKF) to give a prior estimate, ${\hat{x}}_{k}^{-}$, with covariance, $P_{k_{\;4\times 4}}^{-}$. These are then used to approximate the prior estimate as a Gaussian distribution with a mean around ${\hat{x}}_{k}^{-}$. Mathematically, this is defined as:

(11) $$\begin{array}{c} p\left({\hat{x}}_{x}^{-}|z_{k-1}\right)\approx N\left({\hat{x}}_{k}^{-},P_{k}^{-}\right) \end{array}$$

**Figure 1:**
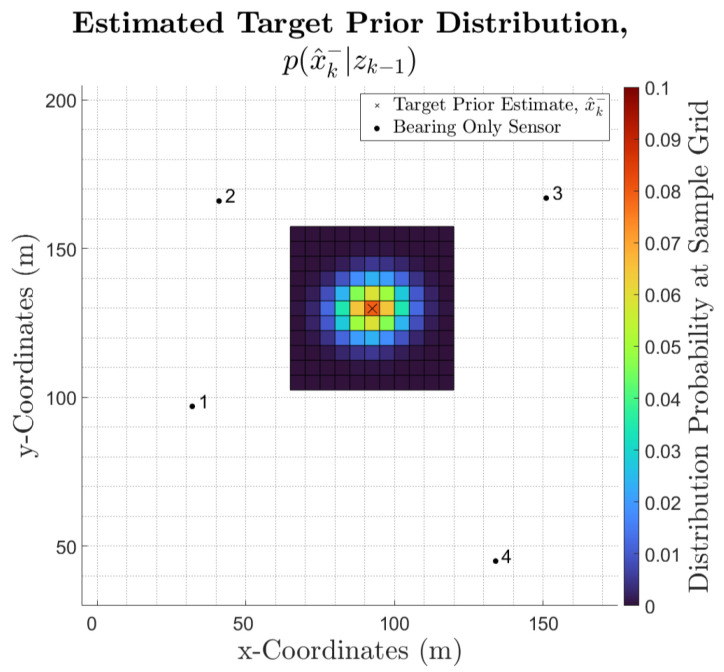
Discretisation of the prior distribution estimate, $p\left({\hat{x}}_{x}^{-}|z_{k-1}\right)$, of a single UAV target, for a WSN scenario consisting of four bearing-only, omnidirectional sensors.

The prior distribution is then discretised into a square-shaped grid, centred around ${\hat{x}}_{k}^{-}$, as shown in Figure 1. The area covered by the discretised sample is determined by the number of samples and the sample resolution (the size of each individual grid), which can each be tuned.

In this example, an 11×11 sampling grid was used, thus generating a sample size of 121. The dimensions of each sample grid were set to 5 m. The selection of these tuning parameters considered the size of the prior distributions to ensure that the higher probability regions are completely included within the discretised area. This was also considered for the sample size and the sample grid size while also taking into account the associated computation cost.

It is noted that a higher resolution and sample size will result in a more accurate discretisation but a higher computational load. On the other hand, a lower resolution and sample size will reduce computational load but will result in low-accuracy discretisation. This trade-off must be determined by the WSN’s characteristics and objectives.

**Figure 2:**
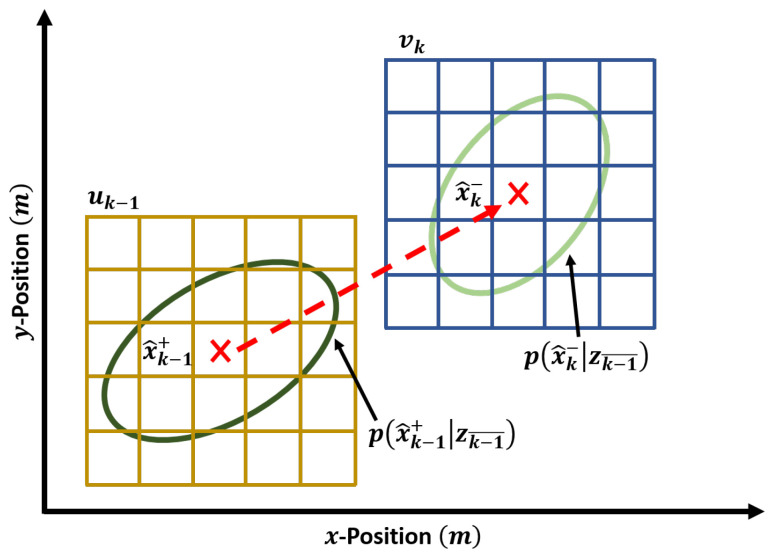
Scenario example for discretising the target distributions. The lower left discretised area, $u_{k-1}$, encompasses the posterior distribution for time step k−1, $\left(p\left({\hat{x}}_{k-1}^{+}|z_{k-1}\right)\right)$, whereas the top right discretised area, $v_{k}$, covers the prior distribution for time step *k*, $\left(p\left({\hat{x}}_{k}^{-}|z_{k-1}\right)\right)$. Note that $u_{k-1}$ and $v_{k}$ have identical sample sizes and resolutions.

Once the prior distribution has been calculated, a new discretised sample space can be drawn from $p\left({\hat{x}}_{k}^{-}|z_{k-1}\right)$ using the pre-defined grid sampling methodology. This new sampling space is referred to as $v_{k\;g_{x}\times g_{y}}$ and will be used to estimate the target posterior distribution for time step *k*. Note that the discretised sample size of $v_{k}$ must be the same as that of $u_{k-1}$. This discretisation process is illustrated in Figure 2.

*Sensor Measurement Marginal Likelihood*

Since this technique predicts sensor measurements as opposed to collecting them, the bearing-only sensor likelihood can no longer be defined as the measurement plus or minus (±) the sensor noise model. For example, should a sensor measurement be available, the sensor likelihood for a bearing-only sensor can be defined as:

(12) $$\begin{array}{c} L_{ki}\left({\hat{x}}_{k}^{-}|z_{k}^{i}\right)=p\left(z_{k}^{i}|{\hat{x}}_{k}^{-}\right)\approx N\left(z_{k}^{i},\sigma_{i}^{2}\right) \end{array}$$

where $p\left(z_{k}^{i}|{\hat{x}}_{k}^{-}\right)$ is approximated by a Gaussian distribution with mean $z_{k}^{i}$, equal to sensor *i*’s measurement, and variance $\sigma_{i}^{2}$, equal to the sensor’s noise model. Note that the ‘equivalence relation’ rule has been applied to Equation (12) [41]:

(13) $$\begin{array}{c} L_{ki}\left(A|B\right)=p\left(B|A\right) \end{array}$$

However, since no sensor measurements are available as a reference point, we can assume that a measurement, $z_{k}^{i}$, can be made anywhere across the discretised grid space, $v_{k}$, with a certain probability.

Therefore, for each discretised grid sample, $v_{k}^{a}$, its centre point, ${\left[v_{k}^{a_{x}},v_{k}^{a_{y}}\right]}^{T}$, is assumed as a possible sensor measurement prediction, ${\hat{\theta }}_{v_{k}^{a}}^{i}$, equal to the bearing from sensor *i* to ${\left[v_{k}^{a_{x}},v_{k}^{a_{y}}\right]}^{T}$, and is treated as a possible value for ${\hat{z}}_{k}^{i}$. Thus, for a bearing-only sensor, ${\hat{\theta }}_{v_{k}^{a}}^{i}$ is calculated as:

(14) $$\begin{array}{c} {\hat{\theta }}_{v_{k}^{a}}^{i}=\arctan\left(\frac{v_{k}^{a_{y}}-i_{y}}{v_{k}^{a_{x}}-i_{x}}\right) \end{array}$$

For each value of ${\hat{\theta }}_{v_{k}^{a}}^{i}$, a Gaussian distribution with a mean at the centre of each discretised grid sample can be calculated using the sensor noise model. This distribution is referred to as the sensor marginal likelihood for each potential measurement prediction. Using the rule in Equation (13), each marginal likelihood can be calculated as:

(15) $$\begin{array}{c} L_{ki}\left({\hat{x}}_{k}^{-}|{\hat{z}}_{k}^{i}={\hat{\theta }}_{v_{k}^{a}}^{i}\right)=\hat{p}\left({\hat{z}}_{k}^{i}={\hat{\theta }}_{v_{k}^{a}}^{i}|{\hat{x}}_{k}^{-}\right)\approx N\left({\hat{\theta }}_{v_{k}^{a}}^{i},\sigma_{i}^{2}\right) \end{array}$$

where each marginal likelihood is approximated as a Gaussian distribution with a mean, ${\hat{\theta }}_{v_{k}^{a}}^{i}$, and variance, $\sigma_{i}^{2}$.

Therefore, in the example discussed, each sensor calculates 121 marginal likelihood, one for where each of the discretised grid samples is considered to be the mean for the sensor model.

**Figure 3:**
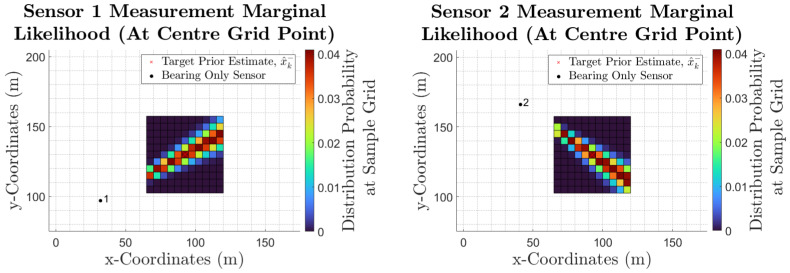
The cone-shaped bearing-only marginal likelihoods for sensors 1 and 2, when the center grid sample is considered to be the predicted bearing measurement.

Figure 3 shows the marginal likelihoods for sensors 1 and 2 when the centre grid sample is considered to be the predicted measurement. Here, the bearing-only cone-shaped sensor model centred around the predicted measurement can be seen. Moreover, a slightly wider distribution can be observed for sensor 1 (up to six pixels wide as opposed to five) due to the target estimate and discretisation area being located further away from sensor 1.

*Sensor Predicted Likelihood Function*

To then obtain a sensor’s predicted likelihood function, $\hat{p}\left({\hat{z}}_{k}^{i}|{\hat{x}}_{k}^{i}\right)$, all its marginal likelihoods must be summed together. This provides the sensor’s overall likelihood of detecting a target at each discretised grid sample, $v_{k}^{a}$. A sensor’s predicted likelihood function is therefore calculated as:

(16) $$\begin{array}{c} \hat{p}\left({\hat{z}}_{k}^{i}|{\hat{x}}_{k}^{i}\right)=\sum_{v_{k}\epsilon S\left({\hat{x}}_{k}^{-}\right)}L_{ki}\left({\hat{x}}_{k}^{-}|{\hat{z}}_{k}^{i}={\hat{\theta }}_{v_{k}^{a}}^{i}\right) \end{array}$$

Please note that since the distribution $\hat{p}\left({\hat{z}}_{k}^{i}|{\hat{x}}_{k}^{i}\right)$ is a summation of all marginal likelihoods for a specific sensor at each discretised sample point, $\hat{p}\left({\hat{z}}_{k}^{i}|{\hat{x}}_{k}^{i}\right)$ is already discretised.

**Figure 4:**
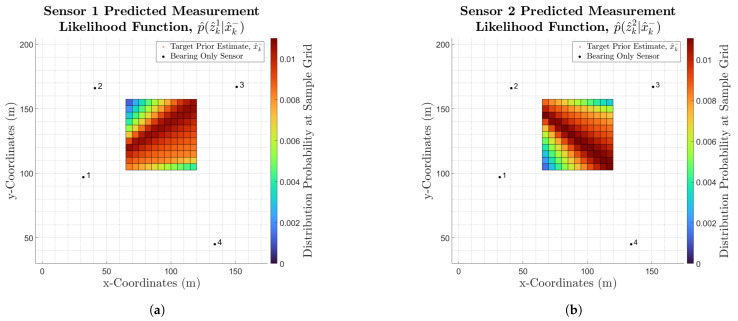
(**a**) Sensor 1’s predicted likelihood function. (**b**) Sensor 2’s predicted likelihood function.

Figure 4a,b show the predicted likelihood functions, $\hat{p}\left({\hat{z}}_{k}^{i}|{\hat{x}}_{k}^{i}\right)$, for sensors 1 and 2 respectively. As in the marginal likelihoods, sensor 1 (Figure 4a) shows a wider region of higher probabilities due to the fact that it is located further away from the target estimate and the discretised area of interest. Moreover, note that the bearing-only marginal likelihood is symmetrical along the distribution mean, but when all marginal likelihoods for a sensor are summed together to obtain the sensor likelihood (Equation (16)), this symmetry is no longer present. Nonetheless, the distribution’s compactness is still range-dependent, following a cone-shaped bearing-only model with a mean skewed by the prior distribution.

*Predicted Posterior Distribution*

Once obtaining the target prior estimate and the predicted sensor likelihood, the target’s predicted posterior distribution, $\hat{p}\left({\hat{x}}_{k}^{+}|{\hat{z}}_{k}^{i}\right)$, for a single sensor can be calculated as:

(17) $$\begin{array}{c} \hat{p}\left({\hat{x}}_{k}^{+}|{\hat{z}}_{k}^{i}\right)=C\times \hat{p}\left({\hat{z}}_{k}^{i}|{\hat{x}}_{k}^{-}\right)\times p\left({\hat{x}}_{k}^{-}|z_{k-1}\right) \end{array}$$

where ${\hat{x}}_{k_{\;4\times 1}}^{+}$ is the target posterior state at time step *k* and *C* is a normalising constant, calculated as the discretised reciprocal of the numerator of Bayes’ rule:

(18) $$\begin{array}{c} C=\frac{1}{\sum_{v_{k}\epsilon S\left({\hat{x}}_{k}^{-}\right)}\hat{p}\left({\hat{z}}_{k}^{i}|{\hat{x}}_{k}^{-}=v_{k}^{a}\right)\times p\left({\hat{x}}_{k}^{-}=v_{k}^{a}|z_{k-1}\right)} \end{array}$$

Note that Equation (17) follows the same structure as the Bayesian Recursive Filtering equation derived from Bayes’ rule. However, the important differences between this and Equation (17) are the $\hat{p}$ and the $\hat{z}$, symbolizing that the sensor measurements, likelihood and posterior distribution are now merely predictions based on the target prior and sensor model, which, in other words, now yield a predicted posterior distribution based on a wide possibility of potential measurements from a single sensor.

*Predicted Posterior Distribution for Multiple Sensors*

When calculating the predicted posterior distribution of a multi-sensor combination, further calculation is required before Equation (17), which calculates the combined measurement likelihood of multiple sensors.

First, knowledge of the possible sensor combinations for the nodes of interest is required, where each node combination, $C_{n_{\;1\times N_{\mathrm{Cn}}}}$, contains a set of sensor nodes, and where $N_{C_{n}}$ is equal to the number of nodes in combination $C_{n}$. Then, the predicted measurement likelihood for node combination $C_{n}$ is calculated using the product rule as:

(19) $$\begin{array}{c} \hat{p}\left({\hat{z}}_{k}^{C_{n}}|{\hat{x}}_{k}^{-}\right)=\prod_{i\epsilon C_{n}}\hat{p}\left({\hat{z}}_{k}^{i}|{\hat{x}}_{k}^{-}\right) \end{array}$$

where $\hat{p}\left({\hat{z}}_{k}^{C_{n}}|{\hat{x}}_{k}^{-}\right)$ is combination $C_{n}$’s predicted measurement likelihood, and $\hat{p}\left({\hat{z}}_{k}^{i}|{\hat{x}}_{k}^{-}\right)$ is sensor *i*’s predicted likelihood.

The predicted posterior distribution for combination $C_{n}$ can then be calculated as:

(20) $$\begin{array}{c} \hat{p}\left({\hat{x}}_{k}^{+}|{\hat{z}}_{k}^{C_{n}}\right)=C\times \hat{p}\left({\hat{z}}_{k}^{C_{n}}|{\hat{x}}_{k}^{-}\right)\times p\left({\hat{x}}_{k}^{-}|z_{k-1}\right) \end{array}$$

where *C* is a normalising constant calculated using Equation (18), where $\hat{p}\left({\hat{z}}_{k}^{i}|{\hat{x}}_{k}^{-}\right)$ is substituted with $\hat{p}\left({\hat{z}}_{k}^{C_{n}}|{\hat{x}}_{k}^{-}\right)$.

**Figure 5:**
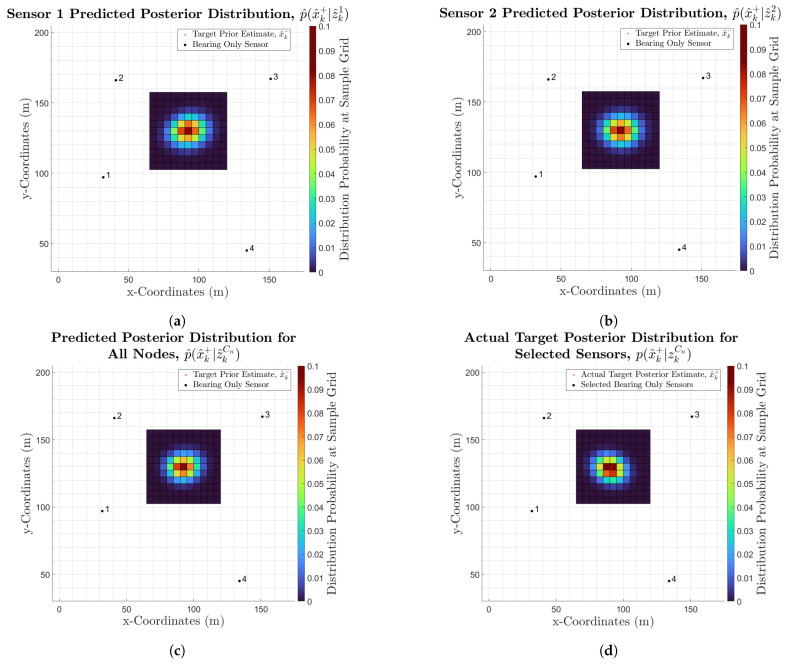
(**a**) Predicted posterior distribution for sensor 1. (**b**) Predicted posterior distribution for sensor 2. (**c**) Predicted posterior distribution for all sensors. (**d**) Actual posterior distribution for the selected sensor combination.

Figure 5a,b show the predicted posterior distributions for sensors 1 and 2 respectively. It can be seen that the angle of the predicted posterior distribution for sensor 1 differs from that of sensor 2, and follows the same direction as its measurement likelihood distribution. Furthermore, the difference in the angles of the predicted posterior distributions for sensors 1 and 2 is due to the fact that for sensor 1, the target is travelling in the same direction as the sensor model’s distribution, resulting in the predicted posterior distribution being along the same axis. Also, since the bearing for sensor 1’s likelihood lines up closely with the prior distribution’s angle (Figure 1), the predicted posterior distribution’s angle also closely resembles that of the prior distribution. On the other hand, for sensor 2, the sensor model’s distribution is roughly perpendicular to the mean bearing of sensor 2’s likelihood, as well as to the target’s direction of travel, resulting in a skewed posterior distribution that is orientated at an angle in between the prior distribution’s angle and the mean likelihood bearing.

Moreover, Figure 5c shows the predicted posterior distribution for the combination with all sensor nodes. A subtle difference in the predicted posterior distribution’s angle for using all sensor nodes is present when compared to that of sensors 1 and 2, where the distribution angle of using all sensors is roughly in between that of sensors 1 and 2. This is expected, as since all four sensors are in a square-like configuration around the target, the predicted distributions of diagonally opposite sensors will be similar. Therefore, when combined, it is expected to have a distribution angle in between that of the individual sensors. Furthermore, it can be seen that the areas around the mean are more compact for the predicted distribution using all sensors, indicating a higher certainty for this prediction.

Once all predicted posterior distributions have been calculated, the expected best node combination can be identified according to the selection criteria, and its nodes are then activated. The actual measurements collected by the activated sensors are then used with the target prior distribution to calculate an actual posterior distribution, $p\left({\hat{x}}_{k}^{+}|z_{k}\right)$ as part of the tracking algorithm. In this work, the ‘Update’ stage of the EKF is used, which outputs a target posterior estimate, ${\hat{x}}_{k}^{+}$, and its posterior covariance, $P_{k_{\;4\times 4}}^{+}$, allowing $p\left({\hat{x}}_{k}^{+}|z_{k}\right)$ to be approximated as a Gaussian with mean, ${\hat{x}}_{k}^{+}$, and covariance, $P_{k}^{+}$. Mathematically, this is defined as:

(21) $$\begin{array}{c} p\left({\hat{x}}_{k}^{+}|z_{k}\right)\approx N\left({\hat{x}}_{k}^{+},P_{k}^{+}\right) \end{array}$$

For visual comparison purposes, in Figure 5d, $p\left({\hat{x}}_{k}^{+}|z_{k}\right)$ is discretised across the sample space for ${\hat{x}}_{k}^{-}$ using:

(22) $$\begin{array}{c} p\left({\hat{x}}_{k}^{+}|z_{k}\right)\;\mathrm{at}\;\mathrm{sample}\;v_{k}^{a}=N\left(v_{k}^{a};{\hat{x}}_{k}^{+},P_{k}^{+}\right) \end{array}$$

where $v_{k}^{a}\epsilon S\left({\hat{x}}_{k}^{-}\right)$.

Figure 5d shows the actual posterior distribution for the selected sensor combination, which uses all nodes. Although the actual distribution is not identical to the shown predicted distribution (Figure 5c), there are still important resemblances between them. For example, the covariance ellipse shape and compactness are comparable. Moreover, the actual distribution’s mean location is very similar to that of the predicted distribution. One difference between the two distributions is the orientation of the distribution. This, as well as the subtle differences in the distribution, are due to the randomness of the noise in the actual measurements, which can never be precisely modelled.

Nonetheless, the above steps have provided a visual validation of the methodology and implementation for predicting the posterior distributions of different sensor combinations, calculated by predicting sensor measurement likelihoods. Moreover, the output from each step of this methodology appears as expected, and the calculated predicted distributions resemble the actual, post-measurement, target posterior distributions.

### 2.2. Sensor Selection Objective Function

Section 2.1 has provided a methodology for predicting the target posterior distribution for different sensor combinations using a sensor model to generate expected measurements. However, in order to make a decision as to which sensor combination is best according to the WSN’s tracking requirements, an objective function is required. This is common to most optimisation techniques, which use a single number to rank sensor nodes.

Therefore, this section details a novel and simplistic objective function that identifies the ‘best’ sensor combination in terms of the smallest cluster size with maintained tracking performance, which are selected based on predicted measurements by considering both the information utility and cost of a sensor combination pre-measurement. This avoids the switching on of all available nodes in the vicinity for an actual measurement, which is known to be a power costly and unnecessary process.

Note that ‘maintained tracking performance’ refers to identical or improved tracking metrics when compared to better but larger clusters. Moreover, slightly worse tracking metrics are accepted on the condition that smaller clusters are established based on objective and not subjective metrics.

#### 2.2.1. Sensor Combination Utility Measure

Since the optimal sensor selection problem for multiple sensors generates multiple predictions for the target posterior distribution, a methodology for calculating and comparing each sensor combination’s information utility is required.

As indicated in Section 1.3, various methods can assign a ‘value of goodness’ to distributions. However, when managing low-cost, bearing-only WSNs, information-theoretic techniques that stem from a distribution’s entropy appear to provide the most efficient optimisation.

Moreover, Shannon Entropy provides a simple, low-cost, single-valued metric that illustrates a distribution’s uncertainty and compactness, which can be easily compared. For this reason, it has been selected as the information utility measure to determine which sensor combination is expected to provide the best tracking performance (utility) within the proposed objective function. Shannon Entropy is defined as [44]:

(23) $$\begin{array}{c} H\left(X\right)=-\sum_{x\epsilon X}p\left(x\right)\times \log_{2}\left(p\left(x\right)\right) \end{array}$$

where X is a set of probabilities.

When applied to this problem, the Shannon Entropy for each combination’s discretised predicted posterior distribution can be calculated as:

(24) $$\begin{array}{c} \Psi_{\mathrm{Utility}}\left(n\right)=H\left[\hat{p}\left({\hat{x}}_{k}^{+}|{\hat{z}}_{k}^{C_{n}}\right)\right]=-\sum_{v_{k}\epsilon S\left({\hat{x}}_{k}^{-}\right)}\hat{p}\left({\hat{x}}_{k}^{+}=v_{k}^{a}|{\hat{z}}_{k}^{C_{n}}\right)\times \log_{2}\left[\hat{p}\left({\hat{x}}_{k}^{-}=v_{k}^{a}|{\hat{z}}_{k}^{C_{n}}\right)\right] \end{array}$$

where $\Psi_{{\mathrm{Utility}}_{\;1\times N_{\mathrm{Cn}}}}$ is the information utility value of combination *n*, $H\left[\hat{p}\left({\hat{x}}_{k}^{+}|{\hat{z}}_{k}^{C_{n}}\right)\right]$ is the Shannon Entropy of combination $C_{n}$’s predicted posterior distribution, which has a range of $\left[0,\log_{2}\left(b\right)\right]$, and where *b* is equal to the discretised sample size of $v_{k}$.

Here, a higher value for $H\left[\hat{p}\left({\hat{x}}_{k}^{+}|{\hat{z}}_{k}^{C_{n}}\right)\right]$ indicates a high uncertainty in combination $C_{n}$’s predicted posterior distribution. In contrast, a lower $H\left[\hat{p}\left({\hat{x}}_{k}^{+}|{\hat{z}}_{k}^{C_{n}}\right)\right]$ value indicates a more certain prediction, thus representing a more compact predicted posterior distribution.

#### 2.2.2. Sensor Combination Cost Measure

In this section, the formulation for calculating the sensor cost for each combination as part of the developed objective function is defined. This is broken down into communication costs and computation costs, as these are the costliest operations during the WSN’s operations.

Note that since this work assumes mains-powered sensor nodes for applications in UTM, the sensor costs do not require an exact calculation in terms of energy consumption. Instead, since an increase in energy consumption is related to an increase in the number of nodes and the node-to-node distance across an active combination, these metrics will be used in the sensor cost calculations to approximate the node energy expense. However, should a requirement for battery-operated nodes with limited energy resources arise, the developed objective function will still be functional with a modification to the calculation of the sensor cost function.

When calculating the communications cost for a combination $C_{n}$, the summation of the squared Euclidean distance between the cluster head and all the nodes in combination $C_{n}$ must first be calculated as:

(25) $$\begin{array}{c} D_{\mathrm{Comms}}\left(n\right)=\sum_{i\epsilon C_{n}}{\left|\left|{\mathrm{CH}}_{k_{x,y}}-i_{x,y}\right|\right|}_{2}^{2} \end{array}$$

where ${\mathrm{CH}}_{k_{x,y_{\;1\times 2}}}$ and $i_{x,y_{\;1\times 2}}$ are the *x*- and *y*-positions of the randomly selected node cluster head, $CH_{k}$, and sensor *i*, respectively, and $D_{{\mathrm{Comms}}_{\;1\times N_{\mathrm{Cn}}}}$ is a vector with length equal to the number of combinations, $N_{C}$, where element *n* contains the summed communication distances for all nodes in combination $C_{n}$.

Equation (25) is defined as the simplified model of the energy expense of radio transmission, where the communication cost between two nodes is proportional to the squared Euclidean distance between them. Note that the distances between nodes are calculated at network creation as these are fixed due to stationary sensing nodes.

However, since the elements of $D_{\mathrm{Comms}}$ will be large in magnitude due to nodes being spaced across a wide area, the communication cost values ($\Psi_{{\mathrm{Comms}}_{\;1\times N_{\mathrm{Cn}}}}$) are normalised by dividing each element of $D_{\mathrm{Comms}}$ by the largest value of $D_{\mathrm{Comms}}$. Note that the combination containing all active nodes will have the largest value of $D_{\mathrm{Comms}}$. By performing this normalisation, $\Psi_{\mathrm{Comms}}$ will always be in the range of $\left[0,1\right]$, where the most expensive combination in terms of communications will have the maximum cost value of 1, and the cheapest combination will have a value of 0.

Therefore, the communications cost for combination $C_{n}$, can be calculated as:

(26) $$\begin{array}{c} \Psi_{\mathrm{Comms}}\left(n\right)=\frac{D_{\mathrm{Comms}}\left(n\right)}{max\left(D_{\mathrm{Comms}}\right)} \end{array}$$

where *n* refers to the combination number of $C_{n}$.

Similarly, the computation cost for using a combination $C_{n}$ is calculated by approximating the total node energy consumption using a computation load metric. This is calculated as the number of nodes in combination $C_{n}$, normalised by the total number of active nodes that can be considered to join a cluster at time step *k*.

Therefore, the computation cost, $\Psi_{{\mathrm{Comps}}_{\;1\times N_{\mathrm{Cn}}}}$, for a combination $C_{n}$ is calculated as:

(27) $$\begin{array}{c} \Psi_{\mathrm{Comps}}\left(n\right)=\frac{N_{C_{n}}}{N_{A}} \end{array}$$

where $N_{C_{n}}$ is the number of nodes in combination $C_{n}$ and $N_{A}$ is the number of active nodes that can join a cluster at time step *k*.

Note that by normalising the computation cost values, $\Psi_{\mathrm{Comms}}$ will also have a range of $\left[0,1\right]$, with the largest combination having a computation cost of 1.

The total cost function for using a combination $C_{n}$ can then be calculated as:

(28) $$\begin{array}{c} \Psi_{\mathrm{Cost}}\left(n\right)=\Psi_{\mathrm{Comms}}\left(n\right)+\Psi_{\mathrm{Comps}}\left(n\right)=\frac{D_{\mathrm{Comms}}\left(n\right)}{max\left(D_{\mathrm{Comms}}\right)}+\frac{N_{C_{n}}}{N_{A}} \end{array}$$

### 2.3. Developed Objective Function

The structure of the developed objective function, $O\left(C_{n}\right)$, follows that of the literature as shown in Equation (1), and is thus defined as:

(29) $$\begin{array}{c} O\left(C_{n}\right)=\alpha \times \Psi_{\mathrm{Utility}}\left(n\right)+\left(1-\alpha \right)\times \Psi_{\mathrm{Cost}}\left(n\right) \end{array}$$

By substituting Equation (28) into Equation (29), the following is then formed:

(30) $$\begin{array}{c} O\left(C_{n}\right)=\alpha \times \Psi_{\mathrm{Utility}}\left(n\right)+\left(1-\alpha \right)\times \left(\Psi_{\mathrm{Comms}}\left(n\right)+\Psi_{\mathrm{Comps}}\left(n\right)\right) \end{array}$$

where $\Psi_{\mathrm{Utility}}\left(n\right)$ is the information utility of combination $C_{n}$, $\Psi_{\mathrm{Comms}}\left(n\right)$ is the communication cost of combination $C_{n}$, $\Psi_{\mathrm{Comps}}\left(n\right)$ is the computation cost for combination $C_{n}$ and α is a defined relative weighting constant which is tuned according to the WSN’s clustering goals.

In Equation (30), the total cost for a combination has been defined as the summation of the combination’s communication and computational costs, as these are equally influenced by the number of nodes within the combination. Furthermore, using the user-defined tuning parameter α, a relative weighting can be given for both the combination’s information utility and costs, depending on the WSN’s clustering goals. For example, if tracking performance is the main requirement, a higher α value should be used to favour information utility, whereas if cluster costs are a main requirement, a lower α value is needed to favour lower cluster costs.

By further substituting Equations (24) and (28) into Equation (30), the sensor selection objective function can be defined in detail as:

(31) $$\begin{array}{c} O\left(C_{n}\right)=\alpha \times H\left[\hat{p}\left({\hat{x}}_{k}^{+}|{\hat{z}}_{k}^{C_{n}}\right)\right]+\left(1-\alpha \right)\times \left(\frac{D_{\mathrm{Comms}}\left(n\right)}{max\left(D_{\mathrm{Comms}}\right)}+\frac{N_{C_{n}}}{N_{A}}\right) \end{array}$$

The sensor combination with the smallest objective function value will be selected as the optimal sensor node cluster, $C_{n_{\;1\times N_{\mathrm{Cn}}}}^{*}$:

(32) $$\begin{array}{c} C_{n}^{*}=\underset{n}{\arg\min}\left[O\left(C_{n}\right)\right] \end{array}$$

It is noted that Equation (32) is constrained to single-hop communication. Moreover, maximum cluster sizes are constrained by node processing capabilities. However, this only becomes problematic for low-cost WSNs with high node densities.

## 3. Performance Analysis of the Sensor Selection Objective Function

In this section, the developed objective function is applied to a WSN in the custom simulation environment to track a single UAV target. To analyse the performance of the proposed sensor selection technique, including the developed objective function’s performance, it is required that a ‘node search’ algorithm is first used to identify possible node combinations for the potential ‘in-range’ nodes. For this, an exhaustive search technique is applied to identify all possible node combinations.

### 3.1. Simulation Parameters

In this section, the simulation parameters used for analysing the developed objective function are stated and summarised in Table 2.

To analyse the performance of the objective function, a WSN scenario of size 200 m × 200 m was configured with 10 omnidirectional low-quality sensors which had a range of 150 m, which, for example, simulate low-cost acoustic sensors. The standard deviation for the sensor noise model, σ, was set to 0.1745 rad, which is equal to ±10°. One UAV target was included in the WSN scenario. The scenarios generated were similar to those displayed in Section 2.1. Furthermore, the simulations were conducted within a custom-built simulation environment in MATLAB and utilised the ‘Sensor Fusion and Tracking Toolbox’.

To track the target, an EKF was used with a constant velocity motion model to provide a target prior estimate and covariance, ${\hat{x}}_{k}^{-}$ and $P_{k}^{-}$, as well as update the estimate with the actual measurements to provide a target posterior estimate and covariance, ${\hat{x}}_{k}^{+}$ and $P_{k}^{+}$. The target state estimate, $\hat{x}$, included the target *x* and *y* positions, as well as the *x* and *y* velocity components. The process noise model for the EKF was set to a diagonal matrix with elements $\left(0.1,0.1,0.14,0.14\right)$. The objective function weighting parameter, α, was set to 0.8, therefore giving the sensor selection an 80% favouring towards a better information utility, $\Psi_{\mathrm{Utility}}$.

Before being able to apply the developed objective function to node combinations, a ‘node search’ algorithm must first identify possible combinations that can be considered. To explore the available in-range nodes and identify all their possible combinations, an exhaustive search technique was used, where the output combinations from this were then examined by the objective function. The formulation for the exhaustive search technique is detailed in Section 3.2 below.

To validate and compare the objective function’s performance, a Monte Carlo simulation was completed with 500 iterations. Here, the actual target location, the prior distribution for time step *k*, and the number of sensors were kept constant for each iteration. However, for each iteration, the sensor locations were changed, thus generating different sensor measurements. Therefore, each Monte Carlo iteration simulated a different scenario for the same time step as opposed to comparing the results from different scenarios that consider a target’s entire trajectory. The purpose of this was to consider and accurately compare the objective function’s versatility for different sensor locations when the target’s prior belief is kept fixed. If the objective function is successful at this, then it will also be successful at tracking a target’s entire trajectory and selecting sensors along its path.

Furthermore, it is also vital to note that, as previously mentioned, limited methodologies where identified which consider predicted posterior distributions. Moreover, as also previously stated, existing sensor selection techniques focus their objective functions on either minimising network costs [7,8], such as energy consumption, without considering the WSN’s goals, which in this case, is target tracking, or by activating all in-range sensors from either existing or dynamic clusters [3]. Thus, this limits the fair direct comparison of this work and the developed objective function with existing techniques.

Therefore, to fairly assess the performance of the developed objective function in meeting its goals of maintained tracking performance with reduced node cluster sizes, the results will be compared to the performance of using all available in-range nodes, where the RMSE (root mean squared error) of the tracking performance and the node cluster sizes will be used as performance metrics. Moreover, the use of an exhaustive search algorithm to identify all the possible node combinations will allow for the global minimum according to the objective function to be identified and selected as the tracking cluster.

### 3.2. Exhaustive Search Methodology

As discussed, to analyse the objective function’s performance, an exhaustive search technique was used to identify all possible node combinations from the nodes within the search area. The methodology for this is detailed below.

First, an initial node selection area is calculated to determine nodes that will be considered to join a cluster. Multiple methods may be used for this. However, in this work, this is determined by the sensor range, and the mean of the target prior distribution, ${\hat{x}}_{k}^{-}$, as sensors outside of this region are statistically unlikely to detect the target, and are thus not required.

**Figure 6:**
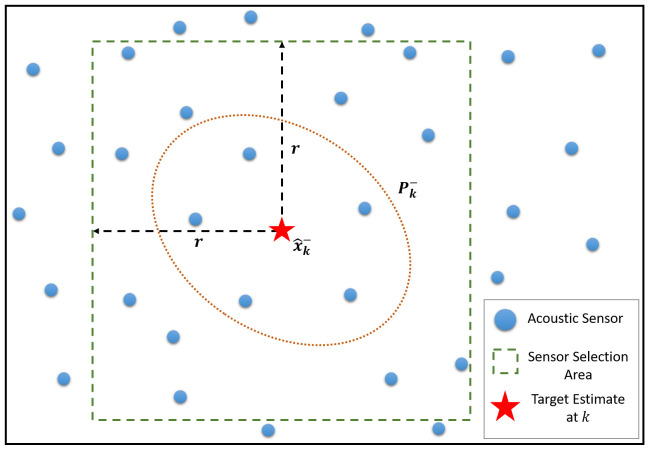
Example scenario of the sensor selection area, where sensors located within the selection square are initially considered to join a cluster.

Here, the sensor range, *r*, may be used to create a selection area centred around the mean of the target’s prior distribution, as shown in Figure 6. This area may be either calculated as a circular area with radius *r*, centred at ${\hat{x}}_{k}^{-}$, or a square centred at ${\hat{x}}_{k}^{-}$ with *x* and *y* coordinate boundaries located at ${\hat{x}}_{k}^{-}\left(1\right)\pm r$ and ${\hat{x}}_{k}^{-}\left(2\right)\pm r$, respectively, where ${\hat{x}}_{k}^{-}\left(1\right)$ and ${\hat{x}}_{k}^{-}\left(2\right)$ are the mean *x* and *y* elements of ${\hat{x}}_{k}^{-}$, respectively.

In this work, the latter technique has been used as since $CH_{k}$ will have a list of all node locations, it can quickly determine which nodes are within the coordinate constraints without further calculation. Moreover, the additional corner regions provided by the square selection area allow nearby sensors that would not have been included by the circular area due to small estimation errors, to still be considered. This is especially useful for scenarios where the target prior distribution is not oriented horizontally or perpendicularly.

Once nodes have been selected for cluster consideration, the non-repeated permutations of these nodes are calculated as the potential cluster combinations. The number of possible node combinations, $N_{C}$, when using an exhaustive search is calculated as:

(33) $$\begin{array}{c} N_{C}=2^{N_{A}}-1 \end{array}$$

where $N_{A}$ is the number of nodes selected to be considered to form a cluster.

After all $N_{C}$ combinations have been determined, the objective function values for all $N_{C}$ combinations are calculated using Equation (31). After calculating these, all $N_{C}$ objective function values are ranked from lowest to highest, with the combination corresponding to the lowest value being selected as the best cluster according to the objective function.

Therefore, using an exhaustive search technique allows for the global minimum to be identified according to the tuned objective function, which relates to the smallest sensor combination that still maintains tracking performance when compared to using all available nodes.

It is important to express that since the exhaustive search technique has a computational complexity of $O\left(2^{n}-1\right)$, increasing the number of nodes, $N_{A}$, exponentially increases the number of possible combinations, $N_{C}$, equally increasing the number of objective function values to calculate. For example, when $N_{A}=10$, $N_{C}=1023$, and when $N_{A}=20$, $N_{C}$ = 1,048,575.

Furthermore, it is noted that since the simulation scenarios used only contained 10 nodes across a small network area of 200 m × 200 m, each with a range of 150 m, all 10 nodes were selected to be considered to form a cluster in each iteration. Thus, the purpose of this initial selection step is more relevant when considering larger node densities across a larger network. Due to the computational complexity of the exhaustive search technique, it was not possible to simulate WSNs with higher node densities across a larger network area due to limitations in both the processing power available and the length of the simulations.

It is for this reason that using an exhaustive search technique to determine the best node cluster according to the objective function is not viable for WSN scenarios with high node densities [32]. However, for the purposes of analysing the objective function’s performance, the use of an exhaustive search method is justified.

### 3.3. Objective Function Performance

In this section, the performance results of the sensor selection objective function using the exhaustive search technique are presented. The simulation parameters detailed in Section 3.1 were used. In line with the objectives of this work, the developed objective function’s performance is analysed using metrics that measure the selected cluster’s size with respect to the number of nodes available, as well as the tracking RMSE (root mean squared error).

In this work, since each Monte Carlo iteration considers a single time step of a different tracking scenario, the RMSE is calculated as the tracking error between the actual target location and the target posterior estimate for each Monte Carlo iteration. A mean RMSE across all the Monte Carlo iterations is then calculated to provide a single-valued tracking performance metric for the different tracking scenarios. The mean values for the Monte Carlo iterations of the different performance metrics are presented in Table 3.

As previously stated, it is also vital to note that limited methodologies were identified that consider predicted posterior distributions. Moreover, identified existing sensor selection techniques focus their objective functions on either minimising network costs using metrics such as energy consumption without considering the WSN’s goals, which, in this case, is target tracking, or by activating all in-range sensors from either existing or dynamically formed clusters.

Reasons for the lack of literature tackling this problem are likely due to the fact that the goal of utilising fewer sensors with maintained tracking performance goes against common intuition, as well as the fact that other techniques do not consider application-specific, non-battery powered WSNs for tracking UAVs across a UTM environment. Thus, this limits the fair direct comparison of this work and the developed objective function with existing techniques. As a result of this, the performance of this work will be compared to the performance of using all available nodes, as it allows for a comparison to a generic and intuitive methodology from which many existing techniques are derived.

**Figure 7:**
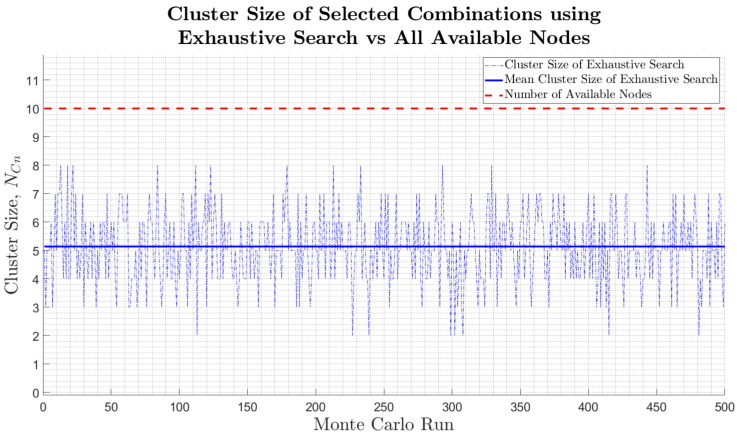
Cluster size of selected combinations using exhaustive search technique and developed objective function, compared to using all available nodes.

In terms of the cluster size reduction, Table 3 illustrates the objective function’s success in reducing the mean cluster size of selected nodes from all available nodes (10) down to a mean of 5.14 nodes. This is a mean cluster size reduction of just under 50%, which is a significant improvement compared to the many existing techniques that select all in-range nodes. This, therefore, also reduces the number of cluster communications occurring and the state estimation computation load at the cluster head by an average of 50%. Figure 7 further illustrates this and indicates cluster size reductions of up to 80%, with just 2 out of the possible 10 nodes being selected to form a cluster at some of the simulation iterations. Furthermore, Figure 7 illustrates that clusters consisting of just 1 node are not formed, which is in line with common intuition to allow for localisation triangulation.

**Figure 8:**
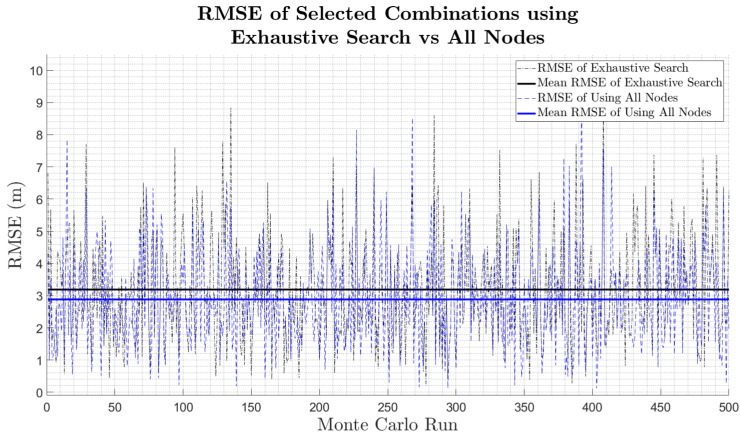
RMSE of selected combinations using exhaustive search technique and developed objective function, compared to using all available nodes.

With respect to the tracking performance of the selected smaller clusters, Table 3 and Figure 8 indicate a small mean RMSE deterioration of just 10.6%, which translates to a mean tracking RMSE difference of just 0.31 m. Furthermore, Figure 8 illustrates that the smaller formed clusters frequently result in a lower RMSE than when using all available nodes.

Therefore, in line with the objectives of this work, the predicted posterior methodology, in conjunction with the developed objective function, can predict an optimal sub-net of available nodes to track a target before collecting a measurement, on average forming clusters with just half of the available nodes. Moreover, these smaller clusters are still able to output comparable tracking performance, with just a 10% deterioration in localisation error. This small deterioration in tracking performance is likely due to the unpredictable random noise present in the measurements of the selected sensors. Nonetheless, due to the resultant large reduction in communication and computation costs during the state estimation process, the slight deterioration in performance is justifiable and acceptable.

## 4. Conclusions

This work has presented a novel methodology to tackle the sensor selection optimisation task for WSNs tracking UAVs in UTM applications before sensor activation and measurement collection has taken place, by using predicted measurement sensor likelihood functions to generate a predicted posterior distribution.

The novelty of this work has been in the development of the objective function, which has been designed in line with the goal of focusing on the WSN’s objective of low-cost, reliable tracking performance as opposed to sensor selection based on node location and/or residual energy levels to increase network lifetime, or the expensive use of all in-range nodes. Moreover, by defining the WSN application of UTM, thus assuming mains-powered sensor nodes, it has enabled the disregard of the energy usage and network lifetime constraints commonly considered within this field. This allowed for the development of a simpler, less costly objective function, which instead approximates the energy usage when calculating a cluster’s cost. Furthermore, the structure of the developed objective function is versatile and allows for different types of WSNs to be considered by adjusting the sensor cost formulations.

The developed objective function aims to optimise the sensor selection problem for the current time step by selecting a smaller sub-net of the available nodes pre-measurement, without compromising the quality of the cluster’s tracking performance. This is completed by considering both the information utility of a sensor combination’s predicted posterior distribution, as well as the cost of obtaining the information. Future extensions to this work may include a mechanism to dynamically adjust how often re-clustering is required based on the target state estimate.

The simulation results indicate that the developed technique can predict an optimal sub-net of available nodes to track a target before collecting a measurement and, on average, forms clusters with just half of the available nodes. Furthermore, the smaller clusters formed can output comparable tracking performance to using all available nodes, with just a 10% deterioration in localisation error. This slight deterioration in tracking performance is thus justifiable and acceptable due to the reduced communication and computation costs occurring during the state estimation process.

However, the use of the exhaustive search technique alongside the developed sensor selection optimisation algorithm results in heavy computation during the combination search process and becomes exponentially unviable for larger WSNs.

Therefore, to further improve the performance of the developed optimisation algorithm, the node combination search process must be greatly improved in order to reduce the number of combination objective functions being calculated. For this, ‘Combinatorial Optimisation’ algorithms such as Branch and Bounds and Genetic Algorithms can be considered in future work, aiming to intelligently search through specific nodes and their combinations, which are statistically more likely to allow for accurate tracking performance. Furthermore, including a dynamic re-clustering time mechanism, which varies how many time steps a cluster is maintained for, depending on the target state estimate, may also be beneficial for further reducing node re-clustering costs.

## Figures and Tables

**Table 1:** The notations and operators used throughout this work.

Notation/Operator	Description
*k*	Time step
*i*	Sensor *i*
${\hat{x}}_{k-1_{\;4\times 1}}^{+}$	Target posterior estimate at k−1
${\hat{x}}_{k_{\;4\times 1}}^{-}$	Target prior estimate at *k*
$P_{k_{\;4\times 4}}^{-}$	Covariance matrix for target prior estimate at *k*
${\hat{x}}_{k_{\;4\times 1}}^{+}$	Target posterior estimate at *k*
$P_{k_{\;4\times 4}}^{+}$	Covariance matrix for target posterior estimate at *k*
$z_{k-1}$	Measurement history until k−1
$z_{k}^{i}$	Sensor *i*’s measurement at *k*
${\hat{z}}_{k}^{i}$	Sensor *i*’s predicted measurement at *k*
$\sigma_{i}$	Sensor *i*’s measurement noise model standard deviation
$CH_{k}$	Cluster head at *k*
$i_{x}$, $i_{y}$	Sensor *i*’s *x*- and *y*-coordinates
*C*	normalising constant for Bayesian recursive filtering equation
$C_{n_{\;1\times N_{\mathrm{Cn}}}}$	Sensor node combination set
$C_{n_{\;1\times N_{\mathrm{Cn}}}}^{*}$	Best sensor node combination set
$N_{A}$	Number of active nodes
$N_{C}$	Number of node combinations
$N_{C_{n}}$	Number of nodes in combination $C_{n}$
$p\left({\hat{x}}_{k}^{-}|z_{k-1}\right)$	Target prior distribution at *k*
$p\left({\hat{x}}_{k-1}^{+}|z_{k-1}\right)$	Target posterior distribution at k−1
$p\left({\hat{x}}_{k}^{-}|{\hat{x}}_{k-1}^{+}\right)$	Target dynamic model distribution
$L_{ki}\left({\hat{x}}_{k}^{-}|z_{k}^{i}\right)$	Marginal likelihood for sensor *i*
$\hat{p}\left({\hat{z}}_{k}^{i}|{\hat{x}}_{k}^{i}\right)$	Sensor *i*’s predicted likelihood function
$\hat{p}\left({\hat{x}}_{k}^{+}|{\hat{z}}_{k}^{i}\right)$	Sensor *i*’s predicted posterior distribution
$\hat{p}\left({\hat{z}}_{k}^{C_{n}}|{\hat{x}}_{k}^{-}\right)$	Combination $C_{n}$’s predicted measurement likelihood
$\hat{p}\left({\hat{x}}_{k}^{+}|{\hat{z}}_{k}^{C_{n}}\right)$	Combination $C_{n}$’s predicted posterior distribution
$u_{k-1_{\;g_{x}\times g_{y}}}$	discretised sample space for $p\left({\hat{x}}_{k-1}^{+}|z_{k-1}\right)$
$v_{k_{\;g_{x}\times g_{y}}}$	discretised sample space for $p\left({\hat{x}}_{k}^{-}|z_{k-1}\right)$
$g_{x}$, $g_{y}$	discretised sample size in *x*- and *y*-directions
${\hat{\theta }}_{v_{k}^{a}}^{i}$	Possible predicted measurement for sensor *i* drawn from $v_{k}$
$\Psi_{{\mathrm{Utility}}_{\;1\times N_{\mathrm{Cn}}}}$	Information utility metric
$\Psi_{{\mathrm{Comms}}_{\;1\times N_{\mathrm{Cn}}}}$	Communications cost metric
$\Psi_{{\mathrm{Comps}}_{\;1\times N_{\mathrm{Cn}}}}$	Computation cost metric
$\Psi_{{\mathrm{Cost}}_{\;1\times N_{\mathrm{Cn}}}}$	Total sensor combination cost metric
$D_{{\mathrm{Comms}}_{\;1\times N_{\mathrm{Cn}}}}$	Total combination communication distance
α	Objective function weighting constant
$N\left(\ldots ,\ldots \right)$	Normal distribution operator
${\left|\left|\ldots \right|\right|}_{2}^{2}$	Squared Euclidean norm operator
$max\left(\ldots \right)$	Maximum value operator
$\underset{n}{\arg\min}\left(\ldots \right)$	Minimum value for imputed argument operator
$diag\left(\ldots \right)$	Diagonal matrix operator

**Table 2:** WSN parameters for the simulation environment to analyse the performance of the sensor selection objective function uing an exhaustive search.

WSN Area Size	200 m × 200 m
Number of Targets, $N_{T}$	1
Number of Sensors, $N_{A}$	10
Sensor Noise, σ	0.1745 rad
Sensor Range, *r*	150 m
Sensor Field of View, FOV	360°
State Estimator	Extended Kalman Filter
State Estimator Motion Model	Constant Velocity
State Estimator Process Noise, Q	$diag\left(0.1,0.1,0.14,0.14\right)$
Objective Function Weighting, α	0.8
Belief discretisation Grid Size	11×11
Belief discretisation Grid Dimensions	5 m × 5 m
Node Search Algorithm	Exhaustive Search
Monte Carlo Iterations	500

**Table 3:** Objective function performance for exhaustive search method compared to using all available nodes (mean metric values across Monte Carlo simulation).

Mean RMSE Using Exhaustive Search	3.19 m
Mean RMSE Using All Nodes	2.88 m
Mean Cluster Size Using Exhaustive Search	5.14
Mean Available Nodes	10
Mean Cluster Size Reduction Using Exhaustive Search	48.62%

## Data Availability

The datasets presented in this article are not readily available as the data is part of an ongoing study.

## Abbreviations

The following abbreviations are used in this manuscript:

WSN	Wireless Sensor Network
UAV	Unmanned Aerial Vehicle
UTM	Unmanned Traffic Management
EKF	Extended Kalman Filter
RMSE	Root Mean Square Error
